# Costs and Length of Stay of Hospitalizations due to Diabetes-Related Complications

**DOI:** 10.1155/2019/2363292

**Published:** 2019-09-08

**Authors:** Ssu-Wei Cheng, Chih-Yuan Wang, Yu Ko

**Affiliations:** ^1^Department of Pharmacy, Shin Kong Wu Ho-Su Memorial Hospital, Taipei, Taiwan; ^2^Department of Pharmacy, College of Pharmacy, Taipei Medical University, Taipei, Taiwan; ^3^Division of Endocrinology and Metabolism, Department of Internal Medicine, National Taiwan University Hospital, Taipei, Taiwan; ^4^Research Center of Pharmacoeconomics, College of Pharmacy, Taipei Medical University, Taipei, Taiwan

## Abstract

**Background:**

Diabetes mellitus (DM) has become a significant worldwide public health problem and economic burden because a great proportion of healthcare costs has been spent on the treatment of DM and its related complications. The aim of this study was to examine the costs and length of stay (LoS) of hospitalizations due to diabetes-related complications in Taiwan.

**Methods:**

This study is a retrospective claim database analysis using the Longitudinal Cohort of Diabetes Patients, with 2012 used as the base year. The hospitalization costs and LoS per admission were estimated for each complication of interest using data from the LHDB 2004 to 2012 cohorts. The presence of eight DM-related complications were identified using the ICD-9-CM codes and procedure codes. ANOVA was used to examine the relationships of diabetes duration with the LoS and costs of the complications.

**Results:**

A total of 27,473 DM patients who were hospitalized in 2012 due to one of the examined DM-related complications were identified. The most common complications that caused the hospitalizations were nonfatal stroke (34.7%) and nonfatal ischemic heart disease (IHD) (28.7%). Amputation was the complication with the longest hospital stay, with a mean ± SD of 21.6 ± 14.1 days, followed by nonfatal stroke (13.6 ± 11.3), ulcer (12.7 ± 11.8), and fatal IHD (12.2 ± 13.6). The complications with the greatest hospitalization cost were fatal IHD (mean = TWD 306,209.8; median = TWD 221,417.0; 1TWD = 0.034USD) and fatal myocardial infarction (mean = TWD 272,840.1; median = TWD 174,008).

**Conclusions:**

This study indicates that DM-related complications are associated with significant hospital LoS and costs. The study results could be useful for economic evaluations of diabetes treatments and the estimation of the overall economic impact of diabetes.

## 1. Introduction

Diabetes mellitus (DM) has become a significant worldwide public health problem and economic burden because a great proportion of healthcare costs has been spent on the treatment of DM and its related complications. Hospital care is usually the *largest component* of medical costs (43% of the total medical costs) of DM in the U.S. [1]. In Canada, when treating acute myocardial infarction in DM patients, acute hospital care accounts for approximately 50% of the first-year healthcare costs [2]. Furthermore, one study in Sweden reported inpatient care costs as high as 57.9% of the total direct medical costs of DM [3]. In Taiwan, a previous study showed that inpatient costs constituted a large part of the total medical costs of DM and its complications, with proportions ranging from 14.6% in patients with an adapted diabetes complication severity index (aDCSI) score of zero to 48.7% in those with an aDCSI score of 5 or greater [4]. In addition, it was found that the greater the number and severity level of DM complications, the higher risk of mortality and hospitalizations [5].

As inpatient costs are likely to account for the majority of overall DM-related medical costs, and DM-related complications are *highly correlated* with hospitalization, it is important to examine the hospitalization costs of DM-related complications. However, previous estimates of the costs associated with the management of DM-related complications in Taiwan have been based on aDCSI scores rather than individual complications. As such, the objective of our study was to examine hospitalization costs and length of stay (LoS) of hospitalizations in Taiwan for each DM-related complication.

## 2. Methods

Ethical approval was obtained from the Shin-Kong Memorial Hospital Institutional Review Board (approval no. 20150712R).

### 2.1. Study Database

In this retrospective database analysis, we used the data from the Longitudinal Cohort of Diabetes Patients (LHDB) 2004 to 2012 cohorts. The LHDB is a database of patients with diabetes who met the following criteria: (1) history of hospitalization for DM or an in-hospital prescription for an antihyperglycemic medication; (2) two or more diagnoses of DM in outpatient settings within one year; or (3) both an outpatient diagnosis of DM and another outpatient visit with a prescription for an antihyperglycemic medication. A cohort of LHDB consisted of 120,000 newly diagnosed DM patients randomly selected each year from the National Health Insurance Research Database (NHIRD) [6], which contained claims data from the compulsory universal healthcare system that covers 99.9% of the Taiwanese population. Among these cohorts of DM patients, those who were hospitalized in 2012 with the primary diagnosis of any complication of interest were included in the analysis, but those who met the following criteria were excluded: (1) aged younger than 18 years; (2) gestational diabetes; or (3) diagnosed with type 1 diabetes mellitus. A patient could be assigned to more than one complication group (i.e., being hospitalized multiple times in 2012 due to different complications). Nevertheless, if a patient was hospitalized for the same complication more than once in 2012, only the first admission was included in the analysis.

### 2.2. Identification of Complication-Associated Hospitalizations

The International Classification of Diseases, Ninth Revision, Clinical Modification (ICD-9-CM) codes and procedure codes were used to identify DM complication-related hospitalizations. The codes associated with the eight common DM-related complications examined in this study are listed in Table 1 [7–9]. A patient was considered to have been hospitalized for a DM-related complication if the primary diagnosis for the admission met the identification codes of that particular complication. A total of eleven complications were under examination: ulcer, amputation, blindness, end-stage renal disease (ESRD), congestive heart failure (CHF), fatal ischemic heart disease (IHD), nonfatal IHD, fatal myocardial infarction (MI), nonfatal MI, fatal stroke, and nonfatal stroke. A fatal event was defined as (1) being hospitalized in 2012 with the primary diagnosis of a complication of interest and a discharge code of “death” or “critically ill and discharged from the hospital voluntarily,” and (2) having no medical claim record in 2013.

### 2.3. Outcome Measures and Statistical Analysis

For hospitalizations due to each DM-related complication, the following outcome measures were assessed: (1) length of stay, (2) total costs of hospitalization, and (3) inpatient drug costs. As only one patient was hospitalized in 2012 as a result of blindness, this complication was excluded from this part of analysis. Baseline characteristics of the selected patients were reported by descriptive statistics. The relationship of DM duration with the LoS and costs of complications was examined by ANOVA.

All monetary values were reported in Taiwan dollars (TWD) (1TWD = 0.034USD). The significance level of this study was set at 0.05. All statistical analyses were performed using SAS software, version 9.4 (SAS Institute, Cary, NC).

## 3. Results

A total of 27,473 DM patients who were hospitalized in 2012 due to one of the study's DM-related complications were identified, and these patients had a total of 29,582 DM complication-related hospitalizations in 2012. The characteristics of the patients are summarized in Table 2. More men than women were hospitalized because of the DM complications (62.5% vs. 37.5%). The mean ± S.D. age of the patients was 68.2 ± 13.6 years, and the duration that these patients had suffered from diabetes was 3.3 ± 2.9 years. In addition, the most common causes of complication-related hospitalizations were nonfatal stroke (34.7%), nonfatal IHD (28.7%), CHF (14.4%), and ESRD (13.9%). The majority (92.9%) of the patients had only one complication-related hospitalization.

For the estimation of costs and LoS per hospitalization, only the patients who were hospitalized for a single complication in 2012 were included in the analysis (*n* = 25,509). As shown in Table 3, amputation was the complication with the longest hospital stay, with a mean ± SD of 21.6 ± 14.1 days, followed by nonfatal stroke (13.6 ± 11.3), ulcer (12.7 ± 11.8), and fatal IHD (12.2 ± 13.6). The complications with the greatest hospitalization costs were fatal IHD (mean = TWD 306,209.8; median = TWD 221,417.0) and fatal MI (mean = TWD 272,840.1; median = TWD 174,008). Similarly, the complications with both the greatest mean and median inpatient drug costs were fatal MI (mean = TWD 29,041.8; median = TWD 11,808.0) and fatal IHD (mean = TWD 26,149.8; median = TWD 11,885.0). In addition, the proportion of drug costs contributing to total hospitalization costs ranged from 3.4% (nonfatal IHD) to 17.9% (ulcer).

The mean LoS, total hospital costs, and inpatient drug costs over different lengths of DM duration for each complication are shown in Table 4. A trend of increasing LoS, total hospital costs, and inpatient drug costs was observed with an increased diabetes duration in nonfatal IHD, nonfatal stroke, congestive heart failure, and end-stage renal disease (*p* < 0.01).

## 4. Discussions

A few studies have examined the economic impact of DM-related complications. Chen et al. [4] reported that the total medical costs, hospitalization costs, and risk of hospitalization all increased with the number and severity of complications. Similarly, Bao et al. [10] also found that both hospitalization costs and LoS were substantially elevated with an increase in the number of complications, with prescribed drugs and laboratory tests being the two major contributors to hospitalization costs. In our study, each DM-related complication's per-episode hospitalization costs and LoS were estimated. The hospitalization cost of one complication could be as high or even higher than the annual health costs of a DM patient without any complication. Furthermore, as our study patients' DM durations were relatively short, and given that a certain complication's hospitalization costs could increase as duration increases, the considerable overall economic impact of DM-related complications cannot be neglected.

In our study, the mean LoS in nonfatal IHD patients was 4.0 days, the shortest hospital stay among the DM-related complications. A significant difference in LoS was reported in a study in Japan where a mean LoS of IHD patients ranged from 10.9 days to 15.1 days depending on the patients' age [11]. The difference could be due to the fact that all hospitals provide care for wound management and nursing home services in Japan, which causes longer hospital stays [11]. On the other hand, in countries other than Japan, during IHD hospitalization, the treatment involves cardiac catheterization (i.e., percutaneous coronary intervention with stent placement), which takes at most three or four inpatient days. As such, the LoS of each DM-related complication and the relative length among the complications could be very different among countries.

In terms of inpatient drug costs, 17.9% of total hospitalization costs of ulcer resulted from drug costs, which proportion was the highest among all DM-related complications, followed by amputation (17.7%). These two complications had similarly high drug cost proportions, and foot ulcers can lead to amputation. In DM patients, the lifetime incidence of foot ulcers could be as high as 25% [12]. Diabetic foot ulcers are susceptible to infection and can cause overwhelming tissue destruction, which is the main reason for the amputation in patients with predominantly neuropathic ulceration. Therefore, it is important to prevent DM-related foot ulcers in order to reduce the incidence of amputation and, in turn, decrease medical expenses.

Bao et al. reported that laboratory tests and prescribed drugs were the two major contributors to hospitalization costs, accounting for 22.4% and 36.2% of the costs, respectively [10]. However, in our study, drug costs of complications contributed only between 3.4% and 17.9% of the total hospitalization costs. The relatively low percentage observed could be because healthcare resource utilization in Taiwan, particularly medication treatments, has been under continual monitoring in the NHI reimbursement program.

There are a few limitations to this study. First, the identification of DM-related complications was based on ICD-9 codes listed in the study claim database, where errors and incompleteness may have existed and resulted in the misclassification of patients in each complication group. However, a previous study evaluating the accuracy of the NHIRD for a DM-related complication has shown high levels of diagnostic accuracy [13]. Second, patients who died of acute incidents such as MI or stroke outside of the hospital had no admission and thusly were not included in the analysis. Third, due to the lack of a nondiabetic control group, in this study it is not possible to discern to which extent the costs and LoS are attributable to diabetes itself. Lastly, despite efforts to find a justifiable definition of death given the study database's restrictions, we were unable to confirm the death of patients and whether the death resulted from a particular complication.

In conclusion, this study indicates that DM-related complications are associated with significant hospital LoS and costs. The study results could be useful for economic evaluations of diabetes treatments and the estimation of the overall economic impact of diabetes.

## Figures and Tables

**Table 1:** Definitions of DM-related complications.

Category	ICD-9 codes [7–9]
Ischemic heart disease (IHD)	Nonfatal events (ICD-9 code 411–414.9)
Myocardial infarction (MI)	Nonfatal myocardial infarction (ICD-9 code 410)
Congestive heart failure (CHF)	ICD-9 codes 428
Stroke	Major stroke (ICD − 9 code ≥ 430 and ≤434.9 or 436)
Amputation	Major limb complications requiring the amputation of a digit or limb for any reason (procedure codes 84.10–84.19)
Blindness	ICD-9 codes 369–369.9
End-stage renal disease (ESRD) [14–16]	Advanced nephropathy (ICD-9 codes 585 and 586) Hemodialysis (procedure code 39.95) or peritoneal dialysis (procedure code 54.98) Renal transplantation (procedure code 55.61 or 55.69)
Ulcer	Chronic ulcer of lower limb (ICD-9 code 707.10)

ICD-9: International Classification of Diseases, 9th edition.

**Table 2:** Characteristics of patients with DM complication-related hospitalizations.

	*n* (%)
Number of patients	**27,473 (100%)**
Number of complication-related hospitalizations	
One	25,510 (92.9)
Two	1,827 (6.7)
Three	126 (0.5)
Four	10 (0.04)
Female	10,307 (37.5)
Age (years), mean ± SD	68.2 ± 13.6
DM duration (years), mean ± SD	3.3 ± 2.9
Region	
North	11,704 (42.6)
Central	6,869 (25.0)
South	7,841 (28.5)
East	958 (3.5)
Islands	101 (0.4)
Number of complication-related hospitalizations	**29,582 (100%)**
Nonfatal stroke	9,532 (34.7)
Nonfatal IHD	7,887 (28.7)
CHF	3,943 (14.4)
ESRD	3,829 (13.9)
Nonfatal MI	2,441 (8.9)
Fatal stroke	839 (3.1)
Amputation	576 (2.0)
Fatal MI	402 (1.5)
Ulcer	68 (0.2)
Fatal IHD	64 (0.2)

SD: standard deviation.

**Table 3:** Costs and length of stay of complication-related hospitalizations (*n* = 25,509).

Complication	LoS (day)		Total hospitalization costs per admission (TWD)		Inpatient drug costs (TWD)		
	Mean (SD)	Median	Mean (SD)	Median	Mean (SD)	Median	Percentage of total hospitalization costs
Nonfatal IHD	4.0 (5.6)	2.0	83,199.3 (89,171.4)	55,574.0	2,816.1 (7,838.2)	1,085.0	3.4%
Fatal IHD	12.2 (13.6)	7.5	306,209.8 (287,206.5)	221,417.0	26,149.8 (34,928.8)	11,885.0	8.5%
Nonfatal MI	8.8 (8.2)	6.0	170,454.6 (141,970.1)	142,171.0	10,349.7 (20,076.3)	3,924.0	6.1%
Fatal MI	10.1 (12.3)	6.0	272,840.1 (313,602.9)	174,008.0	29,041.8 (47,140.3)	11,808.0	10.6%
Nonfatal stroke	13.6 (11.3)	10.0	83,468.6 (101,015.0)	43,307.5	8,574.3 (18,165.1)	2,115.0	10.3%
Fatal stroke	9.9 (9.6)	7.0	147,476.8 (172,818.2)	101,677.0	21,243.4 (60,857.6)	9,462.3	14.4%
CHF	9.6 (9.0)	7.0	63,650.0 (118,867.6)	30,196.0	7,355.9 (20,511.6)	2,225.0	11.6%
Amputation	21.6 (14.1)	18.0	138,573.6 (123,895.0)	99,170.0	24,519.0 (35,566.3)	11,851.8	17.7%
ESRD	11.7 (10.7)	8.0	83,632.5 (99,327.0)	48,907.0	11,293.2 (22,222.7)	3,995.5	13.5%
Ulcer	12.7 (11.8)	8.0	66,978.4 (104,602.6)	31,945.0	11,976.2 (28,528.1)	3,256.0	17.9%

**Table 4:** The relationship between the duration of diabetes and LoS and costs of complications.

Durations (year)	8	7	6	5	4	3	2	1	0	*p* value
LoS (day)										
IHD (nonfatal)	5.06	3.70	3.70	3.23	3.75	3.88	4.11	3.57	3.60	0.002^∗^
IHD (fatal)	11.18	9.75	2.00	29.4	9.00	7.50	7.30	12.83	17.50	0.662
MI (nonfatal)	9.66	8.11	8.78	8.27	7.83	8.63	8.55	7.22	8.86	0.200
MI (fatal)	10.84	12.02	4.07	9.79	13.53	10.58	7.35	8.48	10.68	0.800
Stroke (nonfatal)	15.11	14.52	13.41	12.89	12.90	12.84	12.41	12.45	12.41	<0.001^∗∗^
Stroke (fatal)	10.11	9.86	8.98	10.22	10.09	9.75	10.60	7.83	10.46	0.828
CHF	10.84	9.52	9.77	8.47	9.39	8.93	8.96	9.50	8.78	0.003^∗^
Amputation	22.68	22.29	20.79	22.44	23.67	21.55	20.63	20.38	21.39	0.344
ESRD	13.80	12.72	11.36	10.76	11.15	10.07	10.95	10.59	10.34	<0.001^∗∗^
Ulcer	16.46	16.50	11.82	11.80	17.00	8.08	9.22	11.83	11.75	0.257
Total hospitalization costs per admission (TWD)										
IHD (nonfatal)	105813.2	78756.4	77545.6	70944.3	75617.6	76477.0	84414.0	77650.1	79453.6	<0.001^∗∗^
IHD (fatal)	318628.7	281912.8	310887.5	253998.8	264735.3	275037.0	297469.7	327306.1	383573.8	0.754
MI (nonfatal)	199878.9	149378.3	147066.6	167619.9	152875.9	151352.3	152278.8	146948.9	175779.2	0.172
MI (fatal)	331025.3	186815.5	211844.4	238596.9	362941.8	213620.4	227412.3	221171.4	261033.3	0.663
Stroke (nonfatal)	103200.4	84538.8	79381.5	77218.2	74035.0	74532.4	70144.2	68811.2	68101.5	<0.001^∗∗^
Stroke (fatal)	170386.8	170414.7	117758.0	128903.8	121160.9	116442.3	129253.0	107134.3	128291.4	0.027
CHF	87059.3	60256.4	59106.9	50431.0	55011.7	57651.3	65100.8	57360.7	50313.3	0.001^∗^
Amputation	175860.7	141826.2	124329.0	150628.4	131919.1	118863.8	145931.2	138622.5	139203.4	0.237
ESRD	114526.5	86131.4	77938.2	70208.6	72891.4	71066.0	69233.6	67535.2	67305.0	<0.001^∗∗^
Ulcer	94798.9	175944.5	54184.4	60202.0	94485.8	30818.6	49755.3	43408.3	50690.8	0.053
Inpatient drug costs (TWD)										
IHD (nonfatal)	4657.4	2516.4	2179.4	1800.9	2318.8	2446.9	2818.6	2394.2	2410.5	0.001^∗^
IHD (fatal)	27487.8	6577.9	12033.0	25467.2	30923.0	19864.5	17886.5	33249.5	63543.8	0.119
MI (nonfatal)	14426.4	8137.4	8766.7	7313.6	8546.8	8429.9	7757.4	7723.6	9629.1	0.060
MI (fatal)	37098.2	14468.0	14011.9	25031.7	36284.1	23062.0	18678.6	24064.8	33279.7	0.723
Stroke (nonfatal)	11352.8	8793.2	7391.4	7884.8	7340.9	7196.3	7039.3	6694.7	6223.5	<0.001^∗∗^
Stroke (fatal)	24873.8	25716.0	13527.1	16331.5	17639.2	15157.7	17021.7	12727.5	26114.3	0.614
CHF	10388.5	6580.4	6366.1	4801.2	6584.6	6056.8	7902.4	7099.4	5426.3	0.044
Amputation	29991.1	20134.9	19245.3	21558.0	26379.9	17618.4	25945.0	28481.6	25267.1	0.619
ESRD	16782.7	10243.1	10593.0	9394.4	8756.7	8253.6	8288.9	8088.7	7963.3	<0.001^∗∗^
Ulcer	11832.7	49141.5	6980.2	23677.0	22690.5	1788.2	8632.6	5486.5	6169.5	0.045

^∗^ *p* < 0.01 and ^∗∗^*p* < 0.001.

## Data Availability

The data analyzed for this submission cannot be released to the public.
